# Asthma and Obesity in Children Are Independently Associated with Airway Dysanapsis

**DOI:** 10.3389/fped.2017.00270

**Published:** 2017-12-18

**Authors:** Marcus H. Jones, Cristian Roncada, Morgana Thais Carollo Fernandes, João Paulo Heinzmann-Filho, Edgar Enrique Sarria Icaza, Rita Mattiello, Paulo Marcio C. Pitrez, Leonardo A. Pinto, Renato T. Stein

**Affiliations:** ^1^Laboratory of Respiratory Physiology, Infant Center, School of Medicine, Pontifícia Universidade Católica do Rio Grande do Sul (PUCRS), Porto Alegre, Brazil; ^2^School of Nursing, Centro Universitário Ritter dos Reis, Porto Alegre, Brazil; ^3^School of Medicine, Universidade de Santa Cruz, Santa Cruz do Sul, Brazil

**Keywords:** lung function, asthma, children, overweight, obesity

## Abstract

**Background:**

An increase in the prevalence of overweight and asthma has been observed. Both conditions affect negatively lung function in adults and children. The aim of this study was to analyze the effect of overweight and asthma on lung function in children.

**Methods:**

We designed a case–control study of healthy and asthmatic subjects nested within an epidemiological asthma prevalence study in children between 8 and 16 years of age. The effect of asthma and overweight on lung function was assessed by impulse oscillometry and spirometry obtained at baseline and 10–15 min after salbutamol.

**Results:**

188 children were recruited, 114 (61%) were asthmatics and 72 (38%) were overweight or obese. Children with asthma and overweight had a higher FVC (+1.16 *z* scores, *p* < 0.001) and higher FEV_1_ (+0.79 *z* scores, *p* = 0.004) and lower FEV_1_/FVC (−0.54 *z* scores, *p* = 0.008) when compared to healthy controls. Compared to normal weight asthmatics, the overweight had higher FVC (+0.78 *z* scores, *p* = 0.005) and lower FEV_1_/FVC (−0.50 *z* scores, *p* = 0.007). In the multivariate analysis, overweight was associated with an increase of 0.71 and 0.44 *z* scores in FVC and FEV_1_, respectively, and a reduction in FEV_1_/FVC by 0.40 *z* scores (*p* < 0.01 for all). Overweight had no effect on maximal flows and airway resistance at baseline, and this was not modified by inhalation of a bronchodilator. Asthma was also associated with higher post-BD FVC (0.45 *z* scores, *p* = 0.012) and FEV_1_ (0.35 *z* scores, *p* = 0.034) but not with FEV_1_/FVC and FEF_25–75%_. Two-way analysis of variance did not detect any interaction between asthma and overweight on lung function variables before or after bronchodilator.

**Conclusion:**

Our results suggest that asthma and overweight are independently associated with airway dysanaptic growth in children which can be further scrutinized using impulse oscillometry. Overweight contributed more to the reduction in FEV_1_/FVC than asthma in children without increasing airway resistance. Spirometry specificity and sensitivity for obstructive diseases may be reduced in populations with high prevalence of overweight. Adding impedance oscillometry to spirometry improves our understanding of the ventilatory abnormalities in overweight children.

## Introduction

Asthma and obesity are common chronic diseases that affect the physical, social, and mental health of individuals. Obesity, characterized by the accumulation of body fat, predisposes to several health risks in children (1, 2).

In Brazil, as well as in many countries, the prevalence of obesity in children and adolescents has increased in recent decades (3). Data from Brazilian databases show that 33% of children between 5 and 9 years are currently considered overweight, and 16% of boys and 11% of girls were considered obese (4).

In the same period, there was an increase in the prevalence of asthma in children and adolescents in Brazil (5, 6) with substantial increase in morbidity and health costs (7). Quite consistently, some studies suggest a link between obesity and asthma (8, 9). However, the causal relationship remains controversial and studies exploring the mechanisms involved are required to clarify this association (10–12).

Interestingly, both conditions can negatively affect lung function. In adults, the relationship between overweight/obesity and pulmonary function seems to be well established and most studies detected a reduction in FVC and FEV_1_ in subjects with overweight and obesity (13–15). However, in children, the relationship between overweight and lung function is less clear. Systematic reviews have shown a significant reduction in lung volumes (16), and an increased FVC and FEV_1_ in overweight and obese children (17, 18). Although several studies did not report lower flows (19, 20), some studies have detected a deleterious effect on lung volumes and flows with increased body mass index (BMI) in children (21–24). This pattern of disproportionate but physiologically normal lung growth characterized by an increase in expired volume that is not accompanied by a comparative increase in maximal flows was termed dysanaptic growth (25). More recently, the pooled analysis of several cohorts from healthy and asthmatic children and adolescents confirmed the association of overweight or obesity to higher baseline FVC, TLC, and FEV_1_ and lower maximal flows and FEV_1_/FVC (26). These authors also report an association between the presence of dysanapsis and respiratory morbidity in asthmatic children. Still, most studies show results from baseline lung function only, and it is possible that some degree of bronchoconstriction is present, particularly in children with more persistent asthma. Bronchoconstriction, if present, would amplify the mismatch between FVC and FEV_1_.

In this context, where the preferred parameter to detect obstruction (i.e., FEV_1_/FVC ratio) is similarly affected by either asthma or overweight, the analysis of airway resistance would be an important alternative tool to evaluate whether the observed reduced ratio is an expression of a true obstructive disorder or simply a variant of normality. The aim of this study was to analyze the specific effects of overweight and asthma on lung function in school age children, comparing the results by spirometry and impulse oscillometry, before and after bronchodilation.

## Materials and Methods

### Subjects

We designed a case–control study nested within an epidemiological investigation of asthma prevalence in 8–16 years old children attending public schools in Porto Alegre, Brazil. Briefly, parents responded a respiratory health questionnaire and asthma was defined as a positive answer to all the following questions: “Has your child ever had asthma?” and “Did your child have wheeze in the previous year?” and “Did you use asthma medications in the previous year” (for this question a checklist of specific asthma medications was shown to mothers). Asthmatic children identified by the questionnaire were invited to participate in a lung function study. Classmates without asthma, according to the same questionnaire, were invited to participate in the study as healthy controls. The exclusion criteria were a history of cardiovascular or immune deficiency conditions, presence of other chronic respiratory diseases, recent asthma or rhinitis exacerbations, and diagnosis of an acute respiratory infection or use of oral steroids in the previous month.

### Anthropometric Measurements

Children were weighed with a calibrated scale after removing heavy clothing and shoes, with feet in parallel, head in the midline and arms along the body. Weight was recorded to the nearest 0.1 kg. For the measurement of height, a stadiometer (Altura Exata^®^, Belo Horizonte, Brazil) was used. The measurements of height and weight were used to calculate BMI. *z* scores for BMI and percentiles were calculated using the British 1990 Growth Reference Centiles (27). The classification for normal weight, overweight, and obesity was based on sex- and age-specific cutoff points adopted by the International Obesity Task Force (28). This classification uses centile curves that at age 18 years pass through the widely used BMI cutoff points of 25 and 30 kg/m^2^ for overweight and obesity, respectively. For example, in an 11-year-old child, the BMI cutoff point for overweight is 20.55 for males and 20.74 for females.

### Asthma Classification

Asthma severity was defined by GINA (29) classification standards, based on the treatment required to achieve control of the symptoms. Asthma control was also assessed by a validated Brazilian Portuguese version of the Childhood Asthma Control Test (c-ACT) (30, 31).

### Lung Function Tests

Pulmonary function tests were performed in the Laboratory of Respiratory Physiology by trained professionals, in a quiet environment at room temperature, using an incentive Koko spirometer (Ferraris, USA), and an Impulse Oscillometry System (IOS, CareFusion, Yorba Linda, CA, USA). The order of the lung function tests was IOS, spirometry, bronchodilator, IOS, and spirometry. All evaluations were performed at baseline and 10–15 min after four puffs of salbutamol using a spacer (AeroChamber™), in accordance with the guidelines of the American Thoracic Society and European Respiratory Society (32).

#### Spirometry

The spirometry was performed in accordance with the guidelines published by the ERS/ATS (32). At least three acceptable and reproducible maximal flow–volume curves were obtained before and after using a bronchodilator (Salbutamol, 400 µg). Results were transformed to *z* scores according to international reference values (GLI 2012) that adjust for height, age, ethnicity, and sex (33).

#### Impulse Oscillometry

Measurements were performed and analyzed in accordance with ERS/ATS guidelines (34). Subjects were asked to close their lips around the mouthpiece and cheeks were supported. A nasal clip was used during all the measurements. If artifacts were detected the measurement was repeated until three acceptable curves were obtained. Measurements of respiratory impedance were made at 5 and 20 Hz and the mean values of resistance (R5 and R20) and reactance (X5) from the best three trials were used for the analysis. Results were transformed to *z* scores from reference values (35).

### Statistics and Ethics

Demographic data are presented by descriptive statistics. Continuous data are summarized by arithmetic means and SD, or by median and quartiles. Qualitative and quantitative variables were described, respectively, by mean and SD or median/range or frequency/percentage. Groups were compared by the Student’s *t*-test. Correlation between BMI and lung function variables was tested by the Pearson test. Two-way analysis of variance (ANOVA) and Bonferroni *post hoc* test were used to compare lung function variables in subgroups stratified by overweight and asthma. The effect of overweight and asthma on lung function was evaluated by multiple linear regressions using lung function expressed as *z* scores as the dependent variables, and overweight and asthma as dichotomic independent variables. All tests were two sided and significance was set at the 95% level, and the analyses were performed on SPSS, version 22.0 (SPSS, Chicago, IL, USA). The study was approved by the Human Ethics Committee of the Pontifícia Universidade Católica do Rio Grande do Sul (PUCRS), Porto Alegre, Brazil. Parental written informed consent was obtained from all participants.

## Results

The demographics of the subjects enrolled in the study are presented in Table 1. Of the 188 children, 97 (52%) were females, 114 (61%) had asthma, 49 (26%) were classified as overweight, and 23 (12%) as obese. The BMI *z* score ranged between −2.2 and 3.95. Due to the small number of obese children, all statistical analyses were performed combining all the 72 overweight and obese subjects. The range for age and height were 8.3–14.8 years and 122–171 cm, respectively. Most of the subjects (127/188, 67%) were classified as white. There were no statistical differences between children with asthma and controls regarding anthropometry and ethnicity. Among 114 asthmatic children, reliable information regarding asthma severity and asthma control were obtained in 101 (89%) and 100 (88%), respectively. Children with asthma were classified as having intermittent 39 (39%) or mild persistent asthma (41, 41%) and the remaining 21 (21%) with persistent moderate asthma. None had severe persistent asthma. Regarding asthma control 89 (89%) had partially or uncontrolled asthma in the previous 12 months.

**Table 1:** Clinical and demographic characteristics of the sample.

	Healthy controls, *n* = 74 (39%)	Asthma, *n* = 114 (61%)
Ethnicity (% white)	57 (77)	70 (61)
Sex (% male)	33 (45)	58 (51)
Age (years)	11.17 ± 1.19	11.10 ± 1.10
Weight (kg)	43.69 ± 10.60	44.52 ± 13.29
Weight (*z* score)	0.78 ± 1.00	0.86 ± 1.21
Height (cm)	146.97 ± 8.35	146.55 ± 8.46
Height (*z* score)	0.36 ± 0.92	0.33 ± 0.94
Body mass index (BMI)	19.98 ± 3.46	20.49 ± 4.72
BMI (*z* score)	0.86 ± 1.06	0.90 ± 1.37

### Baseline and Post-BD Lung Function

The results of lung function tests are presented in Table 2, stratified by asthma and by BMI grade. There was no association between asthma severity or asthma control (c-ACT) and BMI or lung function. At baseline, children with asthma had significantly lower FEF_25–75%_ and FEV_1_/FVC and higher R5 and R20 and lower reactance X5 (*p* < 0.01 for all analysis). FEV_1_ was similar in asthmatic and healthy controls. Children with asthma had significantly higher bronchodilator response in FEV_1_ and FEF_25–75%_; 6.6 versus 3.3% (*p* < 0.01) and 23.3 versus 12.2% (*p* < 0.001), respectively. Children with and without asthma had similar reduction in R5 and R20 with bronchodilator.

**Table 2:** Baseline and post-bronchodilator lung function of 188 school age children stratified by asthma and by body mass index (BMI) classification.

	Stratified by asthma		Stratified by BMI classification	
Baseline	Healthy controls (*n* = 74)	Asthma (*n* = 114)	Normal weight (*n* = 116)	Overweight (*n* = 72)
FEV_1_	−0.023 ± 0.906	−0.030 ± 1.051	−0.175 ± 0.907	0.211 ± 1.083**
FVC	−0.014 ± 0.898	0.311 ± 1.096*	−0.074 ± 0.889	0.596 ± 1.117**
FEV_1_/FVC	−0.093 ± 0.746	−0.560 ± 0.884**	−0.238 ± 0.828	−0.600 ± 0.872**
FEF_25–75%_	0.006 ± 0.912	−0.485 ± 1.029**	−0.248 ± 0.990	−0.362 ± 1.047
R5	0.467 ± 0.722	0.852 ± 0.794**	0.636 ± 0.786	0.804 ± 0.783
R20	0.296 ± 0.543	0.643 ± 0.646**	0.542 ± 0.632	0.450 ± 0.625
X5	−0.168 ± 0.927	−0.775 ± 1.930**	−0.482 ± 1.760	−0.623 ± 1.419

**Post-BD**			
FEV_1_	0.188 ± 0.786	0.543 ± 1.134*	0.232 ± 0.901	0.682 ± 1.151**
FVC	0.056 ± 0.879	0.529 ± 1.278**	0.075 ± 0.965	0.781 ± 1.312**
FEV_1_/FVC	0.155 ± 0.700	−0.005 ± 0.770	0.204 ± 0.709	−0.182 ± 0.747**
FEF_25–75%_	0.368 ± 0.797	0.309 ± 0.909	0.375 ± 0.803	0.263 ± 0.960
R5	0.067 ± 0.563	0.305 ± 0.639*	0.191 ± 0.676	0.244 ± 0.522
R20	0.112 ± 0.401	0.381 ± 0.495**	0.310 ± 0.478	0.222 ± 0.475
X5	0.364 ± 0.746	−0.026 ± 1.949	0.066 ± 1.925	0.223 ± 0.859

*All variables expressed as z scores*.

*FVC, forced vital capacity; FEV_1_, forced expiratory volume in 1 s; FEF_25–75%_, forced expiratory flow at 25–75% of FVC; R5, resistance at 5 Hz; R20, resistance at 5 Hz; X5, reactance at 5 Hz*.

*Values expressed as mean ± SD*.

*p < 0.05.

**p < 0.01.

Stratifying the subjects by BMI grade we detected a significant positive effect of overweight on FEV_1_, FVC, and a reduction in FEV_1_/FVC (*p* < 0.01 for all). These differences when compare to non-overweight children were maintained after bronchodilator. Being overweight was not associated with lower maximal flows or higher airway resistance. Overweight children had comparable bronchodilator response in FEV_1_, FEF_25–75%_, R5, R20, and X5 when compared to normal weight children.

### Stratified Analysis of Post-BD Lung Function

**Figure 1:**
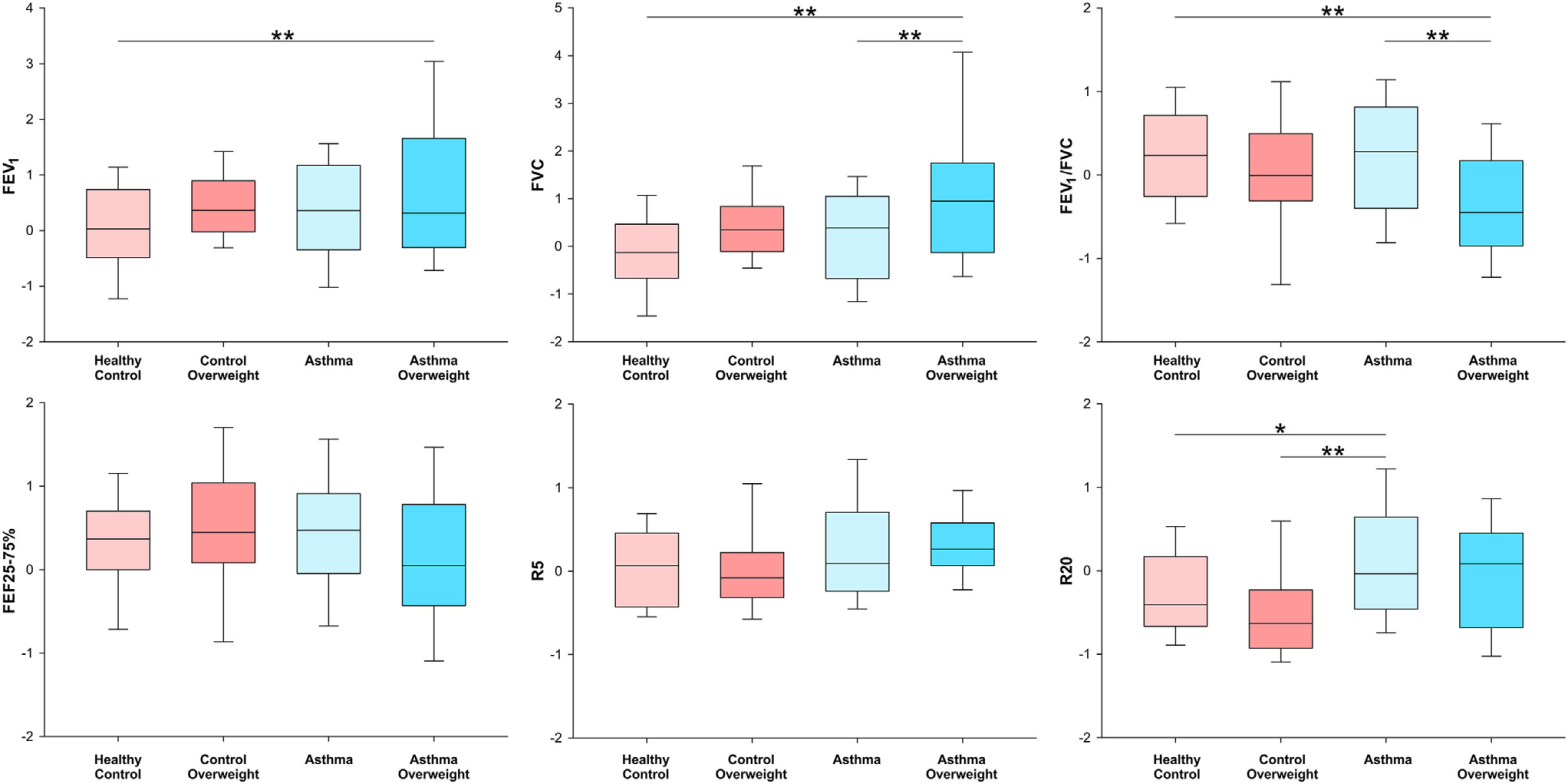
Box-plots showing median and interquartile ranges for FEV_1_, FVC FEV_1_/FVC, FEF_25–75%_, R5, and R20 stratified in four subgroups by the presence of overweight and asthma. *p < 0.05 and **p < 0.01 for analysis of variance with *post hoc* Bonferroni pairwise analysis.

To explore the effects detected in the previous analysis the sample was stratified by asthma and overweight in four subgroups: healthy controls, controls overweight, asthma, and asthma overweight. The analysis was performed on lung function post-bronchodilator to minimize the effect of bronchial tone on the results and is shown in Figure 1. Results are from two-way ANOVA with *post hoc* Bonferroni pairwise analysis. Children with asthma who were overweight had a FVC that was 1.16 *z* scores higher than healthy controls, equivalent of +362 mL or +13.6% (*p* < 0.001). They also had higher FEV_1_ (+0.79 *z* scores, 9.6%, *p* = 0.004) and lower FEV_1_/FVC (−0.54 *z* scores, *p* = 0.008) values when compared to healthy controls. Compared to normal weight asthmatics, the overweight had higher FVC (+0.78 *z* scores, *p* = 0.005) and lower FEV1/FVC (−0.50 *z* scores, *p* = 0.007). The magnitude of the effect of overweight in asthmatic subjects, after adjusting for height, age, and sex was +234 mL (8.7%, *p* < 0.01) in FVC and a reduction of 3.4% in FEV_1_/FVC (*p* < 0.01) when compared to normal weight asthmatic children. Normal weight asthmatics had higher post-BD R20 compared to healthy controls (+0.27 *z* scores, *p* = 0.013) and overweight controls (+0.38 *z* scores, *p* = 0.003).

### Multivariate Analysis of the Effect of Overweight and Asthma on Lung Function

**Figure 2:**
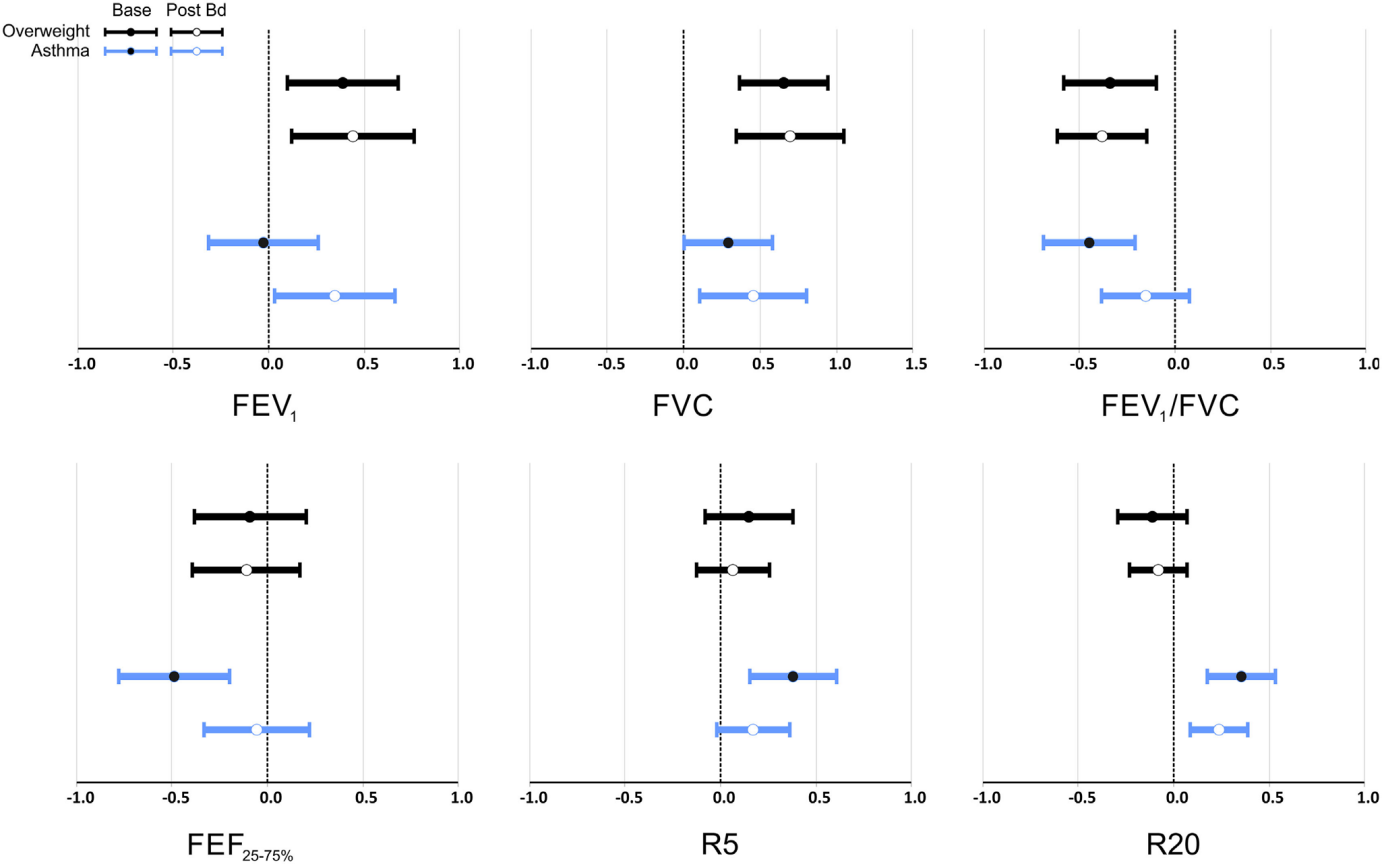
Multivariate analysis of the effect of overweight and asthma on lung function pre- and post-bronchodilator expressed as *z* scores as the dependent variables, and overweight and asthma as dichotomic independent variables. Data is presented as estimated mean and 95% confidence interval of the difference to healthy controls. FEV_1_, FVC, FEV_1_/FVC, and FEF_25–75%_ expressed in *z* scores (33) that adjusts for height, age, sex, and ethnicity; R5 and R20 expressed in *z* scores (35) that adjusts for sex and height.

Figure 2 and Table 3 show the magnitude of the effect of overweight and asthma on lung function estimated by multivariable linear regression, with healthy controls as the reference group. The presence of overweight was associated with an increase of 0.7 *z* scores in FVC, equivalent to 210 mL or 7.5% (*p* < 0.001). FEV_1_ was also significantly higher with overweight, but the magnitude of the difference was smaller in comparison to FVC: +0.39 at baseline and +0.44 *z* scores post-BD (*p* < 0.01 for both). In addition, overweight had a significantly impact on pre- and post-BD FEV_1_/FVC (*p* < 0.001) reducing the ratio by 0.34 and 0.40 *z* scores, respectively. Overweight had no detectable effect on maximal flows and airway resistance at baseline and this was not modified by inhalation of a bronchodilator.

**Table 3:** Multivariate analysis of the effect of overweight and asthma on lung function.

	Baseline						Post-BD					
FEV_1_	Estimated difference	SE	Lower bound	Upper bound	*p*-Value	*R*^2^	Estimated difference	SE	Lower bound	Upper bound	*p*-Value	*R*^2^
Overweight	0.387	0.147	0.097	0.678	0.009	0.036	0.442	0.164	0.119	0.765	0.008	0.073
Asthma	−0.027	0.147	−0.316	0.262	0.855		0.346	0.162	0.026	0.667	0.034	

**FVC**											
Overweight	0.655	0.146	0.366	0.944	<0.001	0.119	0.706	0.180	0.352	1.061	<0.001	0.127
Asthma	0.291	0.146	0.004	0.578	0.047		0.450	0.178	0.099	0.801	0.012	

**FEV1/FVC**											
Overweight	−0.339	0.123	−0.582	−0.097	0.006	0.107	−0.400	0.118	−0.634	−0.167	0.001	0.078
Asthma	−0.449	0.122	−0.690	−0.208	<0.001		−0.135	0.117	−0.367	0.096	0.250	

**FEF_2575%_**											
Overweight	−0.089	0.148	−0.381	0.204	0.550	0.058	−0.127	0.143	−0.409	0.155	0.375	0.006
Asthma	−0.486	0.148	−0.777	−0.195	0.001		−0.041	0.141	−0.320	0.239	0.774	

**R5**											
Overweight	0.148	0.115	−0.078	0.375	0.198	0.066	0.066	0.096	−0.125	0.256	0.497	0.024
Asthma	0.378	0.114	0.152	0.604	0.001		0.171	0.095	−0.017	0.360	0.075	

**R20**											
Overweight	−0.110	0.091	−0.290	0.070	0.229	0.080	−0.079	0.076	−0.229	0.070	0.297	0.066
Asthma	0.352	0.091	0.173	0.531	<0.001		0.238	0.075	0.089	0.386	0.002	

**X5**											
Overweight	−0.110	0.243	−0.589	0.369	0.652	0.034	0.207	0.272	−0.330	0.744	0.448	0.019
Asthma	−0.602	0.242	−1.078	−0.125	0.014		−0.424	0.269	−0.956	0.108	0.118	

*All variables expressed as z scores*.

*FVC, forced vital capacity; FEV_1_, forced expiratory volume in 1 s; FEF_25–75%_, forced expiratory flow at 25–75% of FVC; R5, resistance at 5 Hz; R20, resistance at 5 Hz; X5, reactance at 5 Hz*.

Asthma was also associated with significantly higher FVC, 0.29 *z* scores (*p* = 0.047) at baseline, and 0.45 *z* scores (*p* = 0.012) after bronchodilator. After bronchodilator FEV_1_ was 0.35 *z* scores higher in asthmatics (*p* = 0.034), but not at baseline.

At baseline, asthma was associated with significantly lower FEV_1_/FVC (−0.45 *z* scores, *p* < 0.001), FEF_25–75%_ (−0.40 *z* scores, *p* = 0.001), and higher R5 and R20 (0.38 and 0.35 *z* scores, *p* < 0.001 for both). Reactance was also lower at baseline in children with asthma (−0.6 *z* scores, *p* = 0.014); this difference was reduced and no longer significant after bronchodilator as well as the differences of FEV_1_/FVC, and FEF_25–75%_. After bronchodilator, children with asthma maintained a higher R20 (0.24 *z* scores, *p* = 0.002). Two-way ANOVA did not detect any interaction between asthma and overweight on lung function variables before or after bronchodilator.

## Discussion

Our results suggest that both asthma and overweight are associated with airway dysanaptic growth in children which can be further scrutinized using impulse oscilometry. The assessment of lung function by spirometry and forced oscillations, at baseline and after bronchodilator, allowed the analysis of lung growth and airway growth separately and without the confounding influence of bronchial tone. Our results point toward an independent positive effect of BMI and asthma on FVC and FEV_1_, with a negative effect on FEV_1_/FVC in school age children. However, the discrepancy promoted by overweight and expressed by a lower FEV_1_/FVC is not associated with smaller airways and is also not modified by bronchodilator. Interestingly, after reducing bronchial tone, it becomes clear that the contribution of overweight is substantially bigger than the contribution of asthma in lowering the ratio FEV_1_/FVC (−0.40 versus −0.13 *z* scores, respectively). One could interpret these findings as additional evidence that overweight promotes a lung growth that is different from normal, with a disproportional increase in lung parenchyma (FVC) in comparison to airway (maximal flows and airway resistance). The same pattern of large lungs is seen in swimmers (36), divers (37), and other such athletes, as well as subjects living in high altitudes (38), and these are findings that have no association with respiratory disease. These observations recommend caution to label a lower FEV_1_/FVC as a true obstructive pattern in overweight children. Another implication is that a lower FEV_1_/FVC observed in overweight asthmatic children compared to normal weight asthmatic does not necessarily represent worse clinical respiratory disease.

This is the first study that addresses the impact of overweight and asthma in children comparing two methods that elicit different aspects of lung function, such as spirometry and forced oscillations, before and after bronchodilator. Our findings are in line with studies that reported increased FVC in overweight children (39) but contrast with findings in adults where TLC, FVC, and FEV_1_ were typically reduced (40). However, the effect of weight on lung function seems to be modified by the duration and severity of obesity (41). In addition, an inverse U-shaped association between BMI and FVC in children have been proposed (42). The most plausible explanation for the absence of these ventilatory abnormalities in our sample of school age children is the small number of subjects with obesity.

The finding of increased FVC in children with asthma is interesting and has been reported previously (43, 44). The magnitude of the increase in baseline FVC in our study is similar to that reported for boys in an older study (44) by the order of 3%, and this effect was smaller than the 6.6% reported by Strunk et al. (43). The post-bronchodilator FVC was not reported in these studies, precluding any comparison. Since this increase in FVC is not observed in overweight adults, this finding suggests that it is a developmental abnormality that normalizes, or even reverses by the end of somatic growth as suggested by studies in young adults. Our observation of increased post-BD FEV_1_ is intriguing since most studies have reported lower values in children with asthma when compared to healthy controls (45, 46). Our interpretation is that the potential reduction in airway caliber due to inflammation and remodeling in asthma was offset by the increase in FVC. In our sample, most of the children with asthma were classified as intermittent or mild persistent, unlikely to have significant and persistent reduction in maximal expiratory flows.

Our results do not establish that asthma and overweight as either the cause or the effect of the increase in lung volume, only its cooccurrence. A follow-up study would provide a more accurate description of these associations. Other factors associated with overweight may be relevant for the observed uneven, dysanaptic lung growth. A higher BMI during childhood is associated with higher inspiratory capacity (19), maximal inspiratory pressure (47), and reduced leg length-to-height ratio (48), all presenting some potential to promote disproportional increases in FVC. These factors are not considered in the current predicted equations and could contribute for underestimation of the FVC.

The results of our study suggest that spirometry, which is widely used in the detection and management of asthma, may have lower specificity and sensitivity for asthma in a predominantly overweight society. The observed 13.6% increase in FVC, 7.6% in FEV_1_, and the decrease of 3.5% in FEV_1_/FVC in overweight asthmatics is clinically relevant and would have an impact in spirometry, both at diagnostic level and severity classification. With the increasing prevalence of obesity in children, we will see not only an excess of diagnoses of obstructive disease due to low FEV_1_/FVC but also a delay to recognize a significant reduction in FEV_1_ and FVC due to the use of reference equations not adjusted for body weight. Our data suggest that airway resistance is not increased by overweight and its measurement could be more reliable to assess obstructive diseases in children. Increase in airway resistance was observed in overweight and obese adult subjects (49) but not in preschool children (50). The only study in children that reported an increase in airway resistance with increase in BMI was obtained in children with a history of severe bronchiolitis in the first year of life (51). Our results confirm previous review that airway resistance by IOS can add valuable information in the assessment of respiratory diseases in children (52).

Our study has some limitations that deserve mention. First, the small number of subjects of the study and particularly, the small number of obese and moderate asthmatics subjects enrolled may have reduced power to independently detect the impact of both conditions on lung function. This narrow spectrum was expected from a community-based sample, and it did somehow limit the possible explorations of data in our exploratory study. External validation of the findings, particularly the lack of association of lung function and overweight with clinical outcomes in the asthma group, are needed. In addition, other important variables could not be included in the study; perhaps the most relevant is total lung capacity and residual volume, assessed by plethysmography.

In conclusion, our study demonstrated that both overweight and asthma independently promote an increase in FVC and FEV_1_ with a resulting decrease in FEV_1_/FVC. The results support the concept that overweight promotes an unequal lung growth and that its magnitude is such that it is likely clinically relevant: FVC is 13.6% higher in children with asthma and overweight when compared to healthy controls and 8.7% higher when compared to children with asthma and normal weight. Overweight contributed more than asthma to the deficit in FEV_1_/FVC without increasing airway resistance. Our results suggest that the effect of overweight is mostly in increasing lung size without any perceptible effect on airway resistance; the effect of asthma is both an increase in lung size and in airway resistance. Our results suggest that spirometry specificity and sensitivity for obstructive diseases may be reduced in populations with high overweight and obesity prevalence. Adding measurements of airway impedance by impulse oscillometry to spirometry may improve our understanding of the true clinically relevance of ventilatory abnormalities in overweight children.

## Ethics Statement

This study was carried out in accordance with the recommendations of “Comitê de Ética em Pesquisa – CEP” with written informed consent from all subjects. All subjects gave written informed consent in accordance with the Declaration of Helsinki. The protocol was approved by the “Comitê de Ética em Pesquisa da PontifÍcia Universidade Católica do Rio Grande do Sul.”

## Author Contributions

MJ and RS designed the study. RS, CR, EI, RM, PP, and MF oversaw all clinical aspects of the study (IRB approval, consenting, sample collection). MJ, CR, JH-F, and EI obtained lung function. CR, RM, and EI developed the cloud-based database. MJ, MF, and CR analyzed the data. All authors wrote and reviewed the manuscript.

## Conflict of Interest Statement

The authors declare that the research was conducted in the absence of any commercial or financial relationships that could be construed as a potential conflict of interest.
